# A modified surgical method for the treatment of ONFH: quadratus femoris muscle pedicle bone grafting with preservation of the posterior superior retinacular artery

**DOI:** 10.1186/s12893-022-01834-2

**Published:** 2022-12-22

**Authors:** Changmeng San, Yongqing Xu, Mingjun Lee, Luqiao Pu, Teng Wang, Xiangwen Shi, Siyu Lu, Qi Cheng

**Affiliations:** 1 Kunming Medical University, Kunming, China; 2 Department of Orthopaedics, 920th Hospital of the Joint Logistics Support Force, Kunming, China

**Keywords:** ONFH, Bone grafting, Quadratus femoris muscle pedicle bone graft, Posterior superior retinacular artery, Surgical methods, Blood supply

## Abstract

**Background:**

Osteonecrosis of the femoral head (ONFH) can lead to pain and loss of function of the hip joint, which places a great burden on patients and society. Surgery is the main treatment for osteonecrosis of the femoral head, and quadratus femoris muscle pedicle bone grafting has a definite therapeutic effect as one method of surgery for the treatment of ONFH. However, the posterior superior retinacular artery is often injured during quadratus femoris muscle pedicle bone graft surgery. There is evidence that this artery is extremely important to the femoral head, as injury to this artery will seriously affect the blood supply of the femoral head. Therefore, this situation restricts the clinical application of quadratus femoris muscle pedicle bone grafts. We aimed to explore a new surgical method of quadratus femoris muscle pedicle bone grafting that can preserve the integrity of the posterior superior retinacular artery.

**Methods:**

We modified the traditional quadratus femoris muscle pedicle bone graft and preserved the integrity of the posterior superior retinacular artery. To explore the safety and feasibility of the operation, we simulated the operation on 6 fresh frozen cadavers (12 hips) and measured the related data. We also tried this modified surgical method in the clinic and collected detailed data from the patients.

**Results:**

By simulating the modified quadratus femoris muscle pedicle bone graft on the hip joints of fresh frozen cadavers, we found that the posterior superior retinacular artery existed in all cadaver specimens and that the sources may be different (MFCA or IGA). In the modified operation, the joint capsule did not need to be cut during the operation; therefore, the integrity of the posterior superior retinacular artery was preserved. The quadratus femoris muscle was exposed via the posterior approach of the hip joint, and then the quadratus femoris muscle pedicle bone flap was chiseled. After the pedicle of the quadratus femoris muscle was loosened properly, the migration distance of the quadratus femoris muscle pedicle bone flap reached 5.89 ± 0.45 (χ ± s) cm. The bone flap was trimmed properly and placed on one side. Next, we drilled a bone tunnel from the external intertrochanteric aspect of the capsule of the hip joint, and the bone tunnel broke through the sclerosing zone and proceeded straight to the necrotic area of the femoral head. Next, the necrotic bone was removed with a ring saw and arc bone knife, autogenous bone or allogeneic bone was filled into the bone groove according to the situation, and the cancellous bone in the bone groove was tamped by percussion. Then, the bone flap was inserted into the bone groove, and appropriate pressurization was performed. The depth of the bone groove was determined by the location of ONFH. We found that the furthest distance between the bone groove and the femoral head was 4.76 ± 0.07 (χ ± s) cm and that the length of the bone flap was (4.91 ± 0.23) (χ ± s) cm. This means that when the depth of the bone groove reached the area of ONFH, the quadratus femoris muscle pedicle bone flap had a sufficient length and migration distance to be embedded in the area of ONFH and firmly fixed, and the quadratus femoris did not have much tension. The closest distance between the posterior superior retinacular artery and the bone groove was (1.11 ± 0.96) (χ ± s) cm. When the bone groove was created in this area, the edge of the bone groove had a safe distance of at least 1 cm from the posterior superior retinacular artery of the femoral head. We attempted to implement this modified operation clinically. During the procedure, the quadratus femoris muscle pedicle bone flap was embedded into the drilled bone groove and fixed with a magnesium nail. There was no sliding of the bone flap after the operation, and the posterior superior retinacular artery was intact. We followed the patient for 3 months and found that the patient recovered well with no weight-bearing by the affected limb. The duration of the modified operation was shorter than that of the traditional quadratus femoris muscle pedicle bone graft, the amount of bleeding was significantly reduced, the postoperative pain was lessened, and no special discomfort was reported. Postoperative imaging examination showed that the collapse of the femoral head had been partially corrected and that the bone flap had gradually fused with the surrounding bone.

**Conclusions:**

Through this experimental study, we confirmed the feasibility of the modified method for quadratus femoris muscle pedicle bone grafting with preservation of the posterior superior retinacular artery. This modified operation not only retains the integrity of the posterior superior retinacular artery of the femoral head but also reduces the difficulty of the operation and shortens the surgical time, which is of great clinical significance.

## Introduction

Osteonecrosis of the femoral head (ONFH) is a disorder of the blood supply of the femoral head caused by a variety of factors, which leads to the destruction of the structure of the femoral head and finally develops into collapse of the femoral head, severe pain and dysfunction of the hip joint [1, 2]. Recently, Association Research Circulation Osseous (ARCO) staging [3] has often been used in the stage of ONFH. ARCO staging is recommended for the stage of ONFH, which is of high value in determining the diagnosis and evaluating the therapeutic effect and prognosis. At present, surgery is the main treatment for ONFH, including medullary decompression, bone transplantation, vascular pedicled bone transplantation, osteotomy, porous tantalum rod implantation, stem cells and hip arthroplasty. As a method of vascular pedicled bone transplantation, quadratus femoris muscle pedicle bone grafts have achieved definite results in the clinic, especially for patients with ARCO stage II and IIIa [4, 5]. However, the quadratus femoris muscle pedicle bone graft often causes injury to the posterior superior retinacular artery of the femoral head during the operation, which affects the blood supply of the femoral head itself. Because the posterior superior and inferior retinacular artery is considered to be the main source of blood supply to the femoral head [6], injury to the posterior superior retinacular artery will greatly reduce the blood supply of the femoral head. We intended to improve the surgical method for quadratus femoris muscle pedicle bone grafting and retain the integrity of the posterior superior retinacular artery to reduce the destruction of the blood supply of the femoral head.

The aim of the present study was to accurately evaluate the feasibility of preserving the posterior superior retinacular artery of the quadratus femoris muscle pedicle bone graft to thereby provide an anatomical basis for the clinical application of quadratus femoris muscle pedicle bone grafts to treat osteonecrosis of the femoral head and to develop a new technique for hip-preserving treatment of ONFH.

## Methods

To study the feasibility of preserving the posterior superior retinacular artery in the treatment of avascular necrosis of the femoral head with a quadratus femoris muscle pedicle bone graft, we carried out an autopsy experiment and measured the relevant data during the operation. The purpose of this study was to provide an anatomical basis for the clinical application of femoral quadratus femoris muscle pedicle bone grafts with posterior superior retinacular arteries.

### Materials

Six fresh frozen adult cadavers were used. Integrity of the body, good development of the hip joint, no congenital dysplasia of the hip joint, no hip joint disease and no history of hip joint trauma or surgery were required.

### Procedure

First, the fresh frozen cadavers were thawed and placed on the operating table after thawing was completed. Then, we used the arteria carotis communis and popliteal artery two-way perfusion method to inject latex into the thawed fresh cadavers and began dissection after latex solidification.

**Fig. 1:**
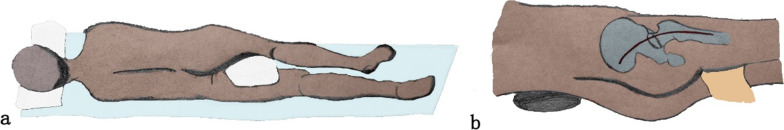
Patient's body position and surgical approach. **a** The patient assumed the lateral recumbent position, the affected side faced upward, flexion of the hip joint and knee joint of the affected limb was nonfixed, and the lower limbs of the healthy side were straightened. **b** The surgical approach started at the posterior approach of the hip joint 5 cm from the posterior superior iliac spine, extended along the direction of the gluteus maximus muscle fiber to the posterior edge of the greater trochanter, turned to the femoral shaft, and then extended 5 cm downward

**Fig. 2:**
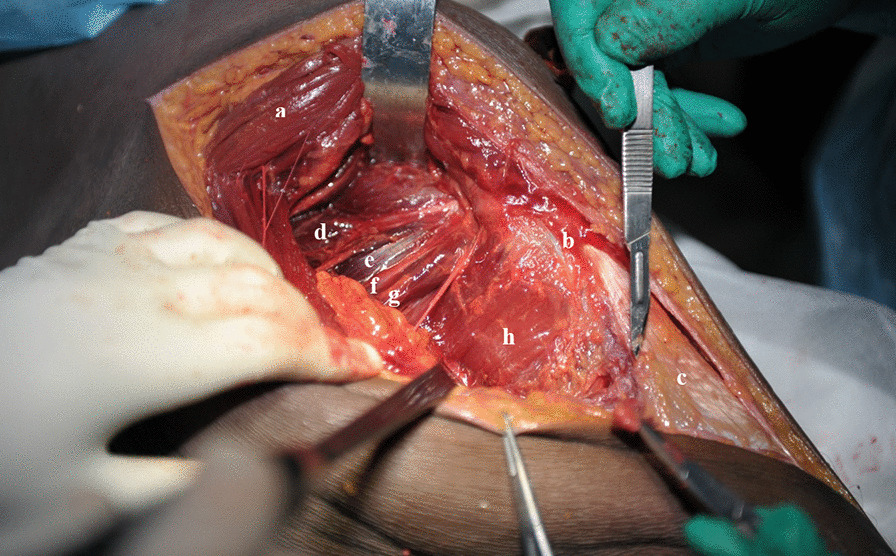
Pulling of the gluteus maximus muscle to separate and expose the external circumflex muscle group. **a** Gluteus maximus; **b** Greater trochanter; **c** Tensor fasciae latae; **d** Piriformis; **e** Superior gemellus; **f** Obturator internus; **g** Inferior gemellus; **h** Quadratus femoris

**Fig. 3:**
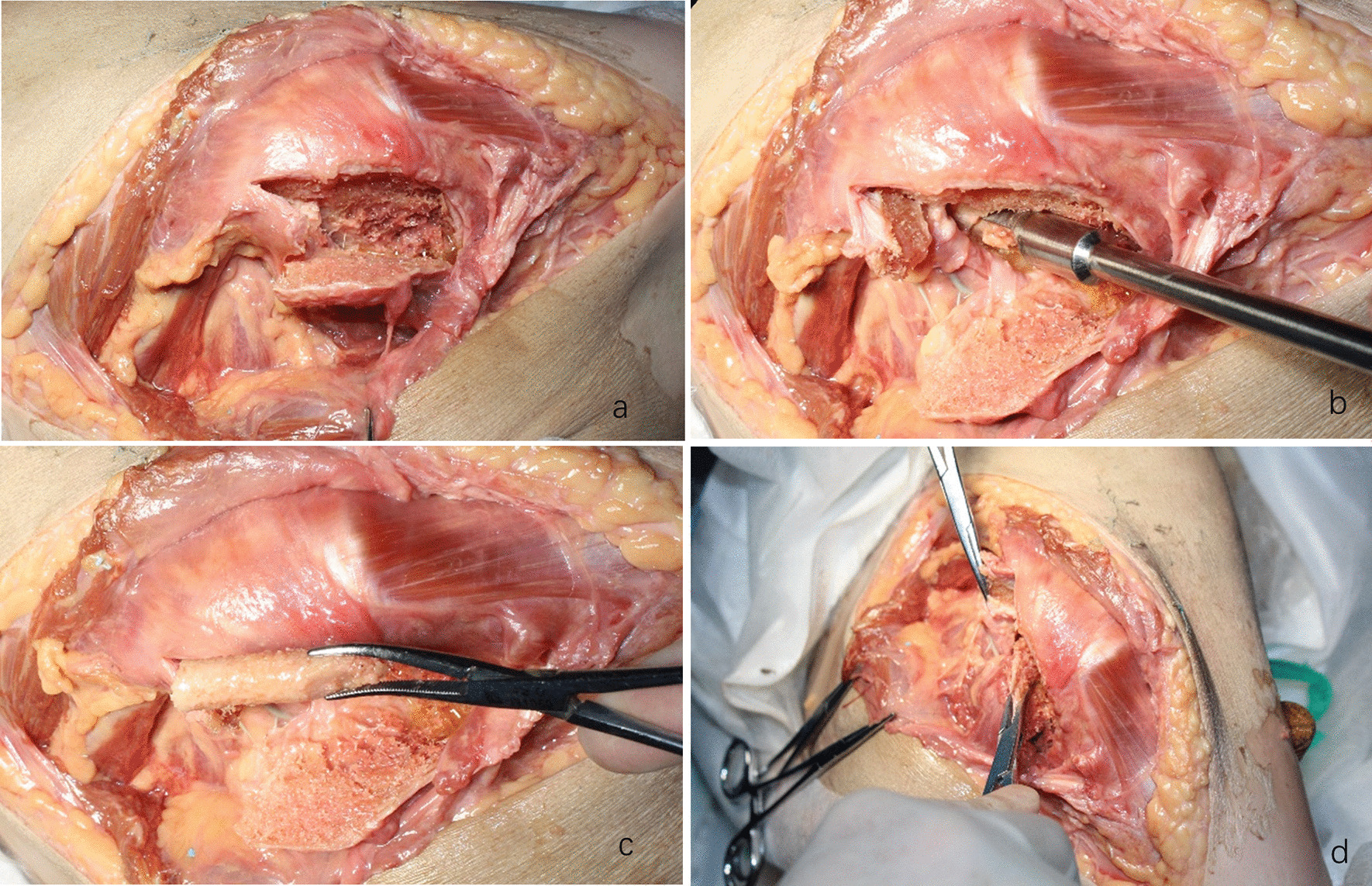
Surgical procedure. **a** Exposure of the quadratus femoris muscle. **b** Chiseling of the bone flap. **c** Drilling of the bone groove. **d** Insertion of the bone flap into the groove

The body was fixed in the lateral recumbent position with the operative side facing upward and the other lower limb in the straight position. Flexion of the hip joint and knee joint on the operative side was nonfixed so that movement in all directions could be carried out during the operation. The surgical approach was the posterior approach of the hip joint. The incision started approximately 5 cm from the posterior superior iliac spine, extended along the gluteus maximus muscle fiber direction to the posterior edge of the greater trochanter of the femur, and then turned to the femoral shaft, extending downward approximately 5 cm. The incision was arc-shaped, with a full length of 10 ~ 15 cm (Fig. 1). To facilitate the anatomy, we cut and separated by layers according to the posterior approach to fully expose the surgical field (Fig. 2).In the deep separation of this area, the sciatic nerve did not need to be separated, which would have easily caused bleeding and postoperative adhesion, but could be pushed to the posterior medial side together with adipose tissue. The internal rotation of the hip joint to tighten the external circumflex muscle could also keep the sciatic nerve away from the surgical area. The next step was to cut off the quadratus femoris muscle pedicle bone flap. Before amputation, the upper and lower boundary of the quadratus femoris and the stopping point of the muscle pedicle was fully exposed. The pedicle of the quadratus femoris muscle was located in the middle of the bone flap as far as possible, and the length of the cut-off bone flap was slightly longer than the stopping point of the quadratus femoris muscle (approximately 4–6 cm), 1.5–2.5 cm in width and 0.5–1.5 cm in depth. When using an osteotome to chisel the bone flap, attention was given to protecting the muscle pedicle of the quadratus femoris muscle to prevent damage to its internal vessels. After the bone flap was chiseled, it was opened laterally together with the quadratus femoris muscle. At this time, the pedicle of the quadratus femoris muscle could be loosened slightly to facilitate the placement of the bone flap, but the loosening was not allowed to be too extensive, in order to avoid damaging the blood supply of the muscle bone flap. The upper margin between the intertrochanteric crest and the capsule stop of the posterior hip joint was used as the entrance and slotted to the femoral neck. The actual direction of the operation was judged according to the direction of the necrotic area. The artery at the base of the femoral neck was avoided when slotting. During the operation, the depth of the groove was appropriate to penetrate the sclerotic zone of the femoral head to reach the necrotic area, and C-arm fluoroscopy helped to adjust the direction and depth. After the necrotic bone was removed, appropriate cancellous bone or allogeneic bone was implanted and compacted according to the actual situation, and then the chiseled bone flap was inserted into the groove and pressed firmly. Flexion and extension of the hip joint indicated that the bone flap was stable without sliding or prolapse, and if it was unstable, the bone flap was fixed with screws. After suturing layer by layer, the operation was completed (Fig. 3).

### Measurements

In the experiment, we tracked the origin and course of the posterior superior retinacular artery of the femoral head. The safe distance between the drilling position of the bone groove and the MFCA and its branches was measured. The related data of the modified quadratus femoris muscle pedicle bone flap were measured. Clinically, we compared the changes before and after the operation through the description and follow-up of a patient with ONFH who received a modified quadratus femoris muscle pedicle bone graft.

## Results

### The quadratus femoris muscle and the bone flap

**Fig. 4:**
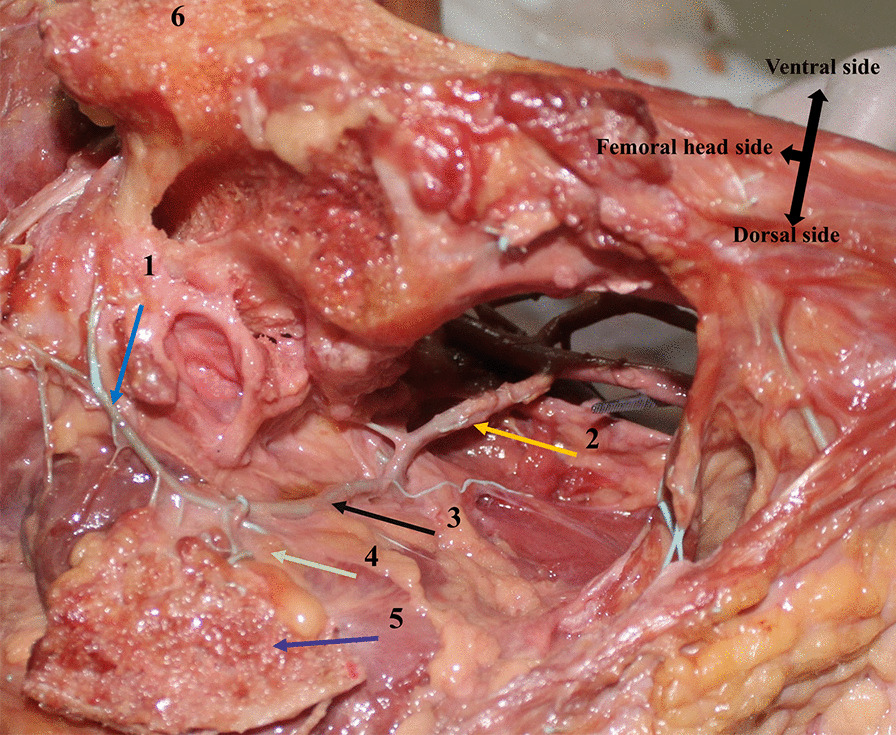
Blood supply of the quadratus femoris muscle. 1. IGA; 2. MFCA; 3. Deep branch of the MFCA; 4. Quadratus femoris muscle; 5. Quadratus femoris bone flap; 6. Greater trochanter

There was no absence of the quadratus femoris muscle in 12 hips. All the quadratus femoris muscles originated from the outside of the ischial tubercle and ended at the intertrochanteric crest. The quadratus femoris muscle is a rectangular short muscle with a length of 3.72 ± 0.30 cm, and the distance between the upper and lower edges of the stopping point is 4.23 ± 0.51 cm. In the anatomy of 12 hips, it was found that the blood supply of the quadratus femoris muscle mainly came from the MFCA (medial femoral circumflex artery) and IGA (inferior gluteal artery) (Fig. 4). After the origin of the MFCA, the deep branch moved backward around the femoral neck on the superior edge of the external obturator muscle to the anterior edge of the external circumflex muscle, ended outward at the trochanter fossa, and sent out the greater trochanter branch near the upper edge of the quadratus femoris stopping point. The deep branch of the MFCA branched backward into the abdomen of the quadratus femoris muscle. The IGA branched into the muscle at the upper edge of the quadratus femoris. The blood supply of the quadratus femoris muscle was very abundant.

**Fig. 5:**
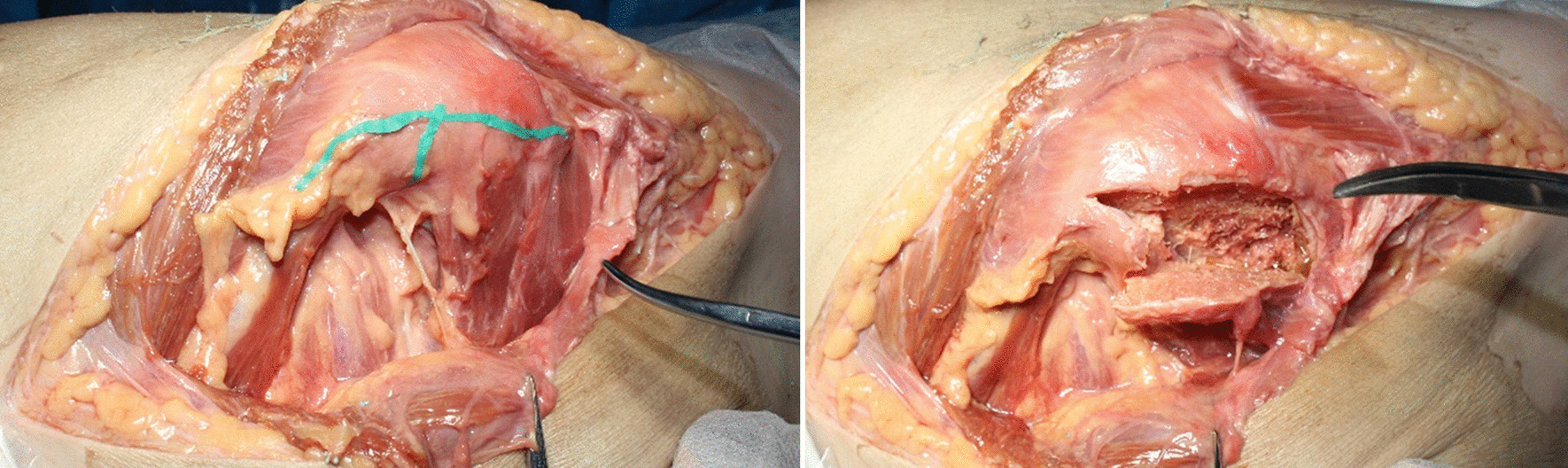
During the osteotomy of the quadratus femoris bone flap, the length of the bone flap was slightly longer than the distance between the upper and lower edge of the quadratus femoris muscle to maximize the blood supply of the bone flap

The blood supply of the quadratus femoris muscle pedicle bone flap comes from a wide range of sources, mainly from the deep branch of the MFCA and the greater trochanter branch of the IGA. When the bone flap is removed, the blood supply of the bone flap can be preserved as much as possible if the edge of the bone flap exceeds the upper and lower edge of the quadratus femoris muscle (Fig. 5). Regarding anatomy, the size of the bone flap was (4.91 ± 0.23) cm in length, (1.64 ± 0.14) cm in width and (0.75 ± 0.15) cm in depth.

### Measurement result

**Fig. 6:**
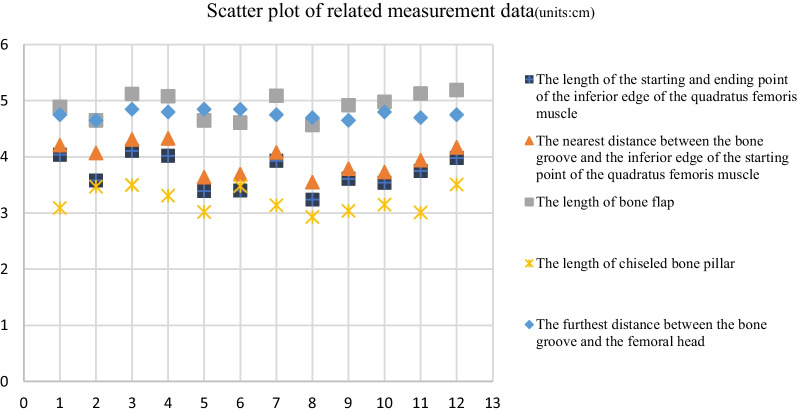
Scatter plot of related measurement data

Our measurements are sorted in the table below (Table 1). To add this information more intuitively, we plotted the data (Fig. 6). The table and figure show that the length of the quadratus femoris muscle was elongated from 3.72 ± 0.30 to 3.96 ± 0.27 cm after the bone flap was embedded in the bone groove. This means that the muscle of the bone flap is slightly tightened after being embedded in the bone groove, but the extent of elongation is small.

**Table 1:** Morphological measurement of quadratus femoris muscle (units:cm)

Hips	A	B	C	D	E
1	4.04	4.21	4.89	3.09	4.75
2	3.58	4.07	4.65	3.47	4.65
3	4.11	4.31	5.12	3.5	4.85
4	4.02	4.33	5.08	3.31	4.8
5	3.39	3.64	4.65	3.02	4.85
6	3.4	3.69	4.61	3.48	4.85
7	3.93	4.08	5.09	3.14	4.75
8	3.24	3.55	4.57	2.93	4.7
9	3.61	3.79	4.92	3.04	4.65
10	3.54	3.73	4.98	3.15	4.8
11	3.75	3.94	5.13	3.01	4.7
12	3.98	4.17	5.19	3.51	4.75
Total	3.72 ± 0.30	3.96 ± 0.27	4.91 ± 0.23	3.22 ± 0.22	4.76 ± 0.07

A. The length of the starting and ending point of the inferior edge of the quadratus femoris muscle. B. The nearest distance between the bone groove and the inferior edge of the starting point of the quadratus femoris muscle. C. The length of bone flap. D. The length of chiseled bone pillar. E. The furthest distance between the bone groove and the femoral head

After measurement, the length of the bone column removed after slotting was 3.22 ± 0.22 cm, and the length of the bone flap was 4.91 ± 0.23 cm. During the operation, after the bone column is removed, the dead bone will be further removed in the bone groove, so the depth of the bone groove is slightly larger than the length of the bone column. The furthest distance from the bone groove to the femoral head was 4.76 ± 0.07 cm. In the removal of dead bone, it is still necessary to retain the cartilage under the femoral head and appropriate bone mass to prevent artificial collapse caused by excessive bone removal. This means that the depth of the bone groove must be less than the furthest distance between the bone groove and the femoral head. Therefore, the length of the bone flap must be larger than the depth of the bone groove, and the result was significantly similar to this. In the process of graft bone compression, the length of the bone flap will be reduced, and with the compression of the bone, the necrotic area of the femoral head that has been cleared will also be propped up by the bone flap.

**Fig. 7:**
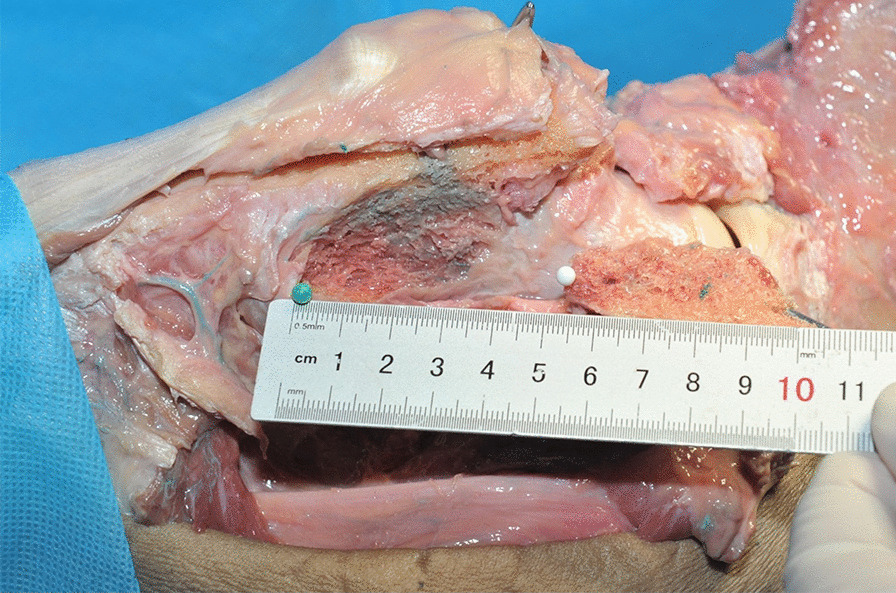
The migration distance of the bone flap muscle

To embed the quadratus femoris muscle pedicle bone flap more deeply into the femoral head, to reduce the tension of the quadratus femoris muscle and to prevent the bone flap from being pulled out, the upper and lower edges of the quadratus femoris muscle can be loosened properly, which in turn can increase the migration distance of the quadratus femoris muscle and make the bone flap embed more deeply. However, attention should be given to protecting the fascial blood vessels of the greater trochanter and the quadratus femoris during release. In this study, after proper release, the migration distance of the quadratus femoris muscle reached 5.89 ± 0.45 cm, which provided a sufficient length for the bone flap to be fully embedded in the femoral head (Fig. 7).

**Fig. 8:**
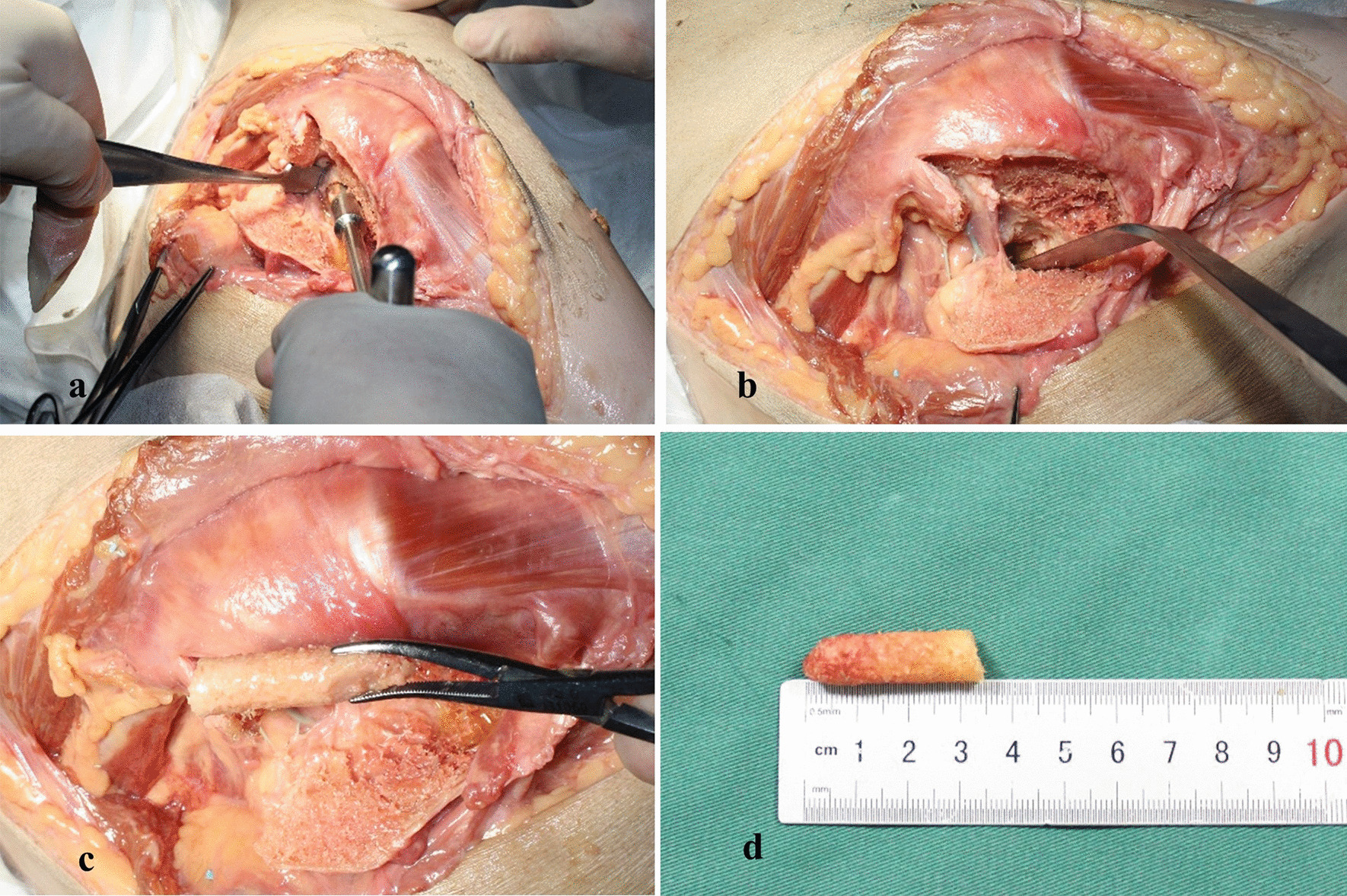
Chiseling of the quadratus femoris bone flap (right hip). **a** Drilling with a ring saw toward the femoral head. **b** The entry point of the bone groove at the outside of the hip capsule at the posterior base of the femoral neck **c** A bone column removed by a ring saw in the bone groove. **d** Measurement of the length of the bone column

After chiseling the bone flap, the posterior side of the femoral neck was slotted to insert the bone flap. We set the entry point of the bone groove at the outside of the hip capsule at the posterior base of the femoral neck, so there was no need to cut open the joint capsule. Osteotome and ring saws (diameter of 10 mm) were selected to make the bone grooves commensurate with the size of the bone flap. After the bone groove was chiseled, the dead bone was removed from the necrotic area at the distal end of the bone groove and the necrotic area around the bone groove with curved bone knives and other instruments with a certain radian. In anatomical practice, we used a ring saw to simulate the chiseling of bone grooves (Fig. 8).

**Fig. 9:**
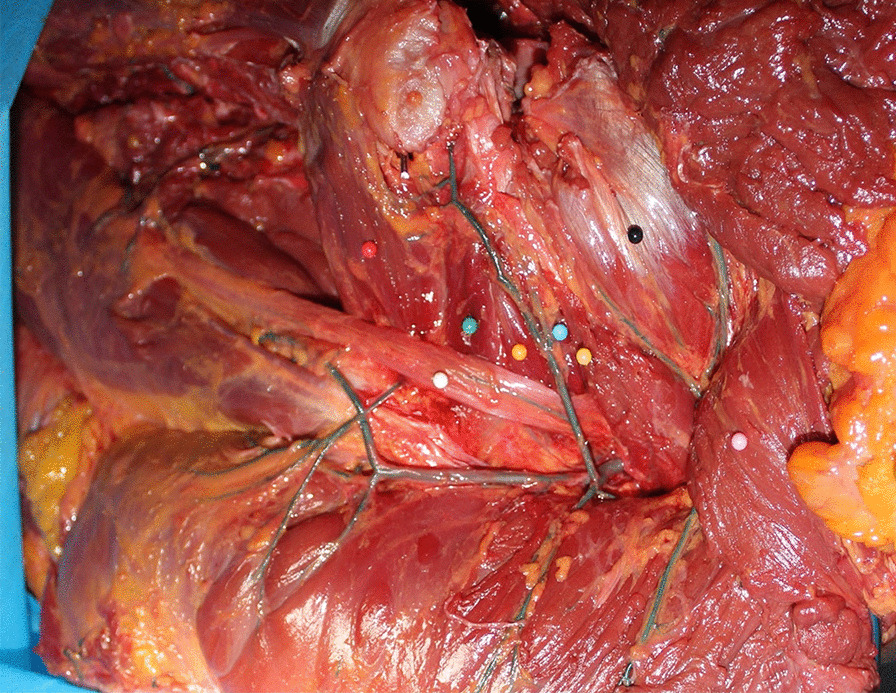
The position of the origin of the posterior superior retinacular artery. It can be seen that the branches of the IGA enter the hip joint capsule. Black ball: gluteus minimus, pink ball: gluteus medius, white ball: sciatic nerve, yellow ball: piriformis muscle, blue ball: superior quadratus muscle, orange ball: obturator muscle, green ball: inferior quadratus muscle, red ball: quadratus femoris

**Fig. 10:**
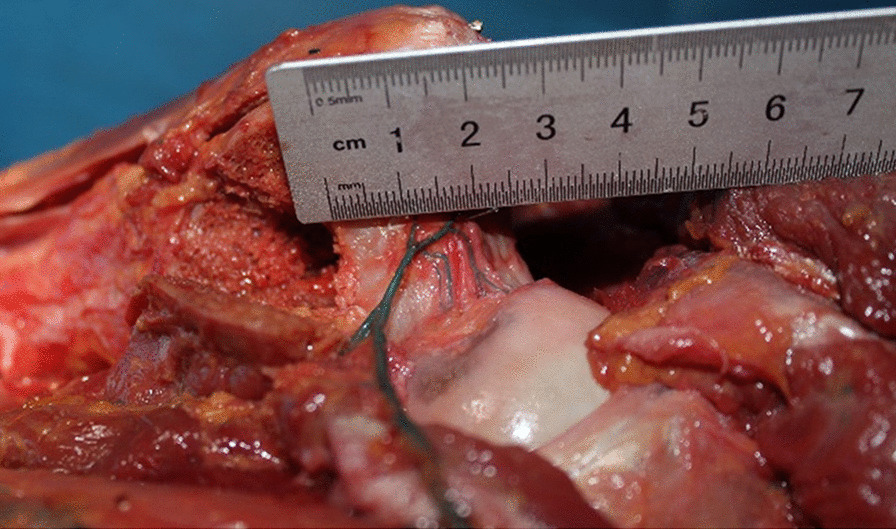
The distance between the bone groove and the posterior superior retinacular artery

In the experiment, it was found that most of the retinacular artery of the femoral head originated from the deep branch of the MFCA and the transverse branch of the LFCA (10 hips), which formed an arterial ring at the base of the femoral neck and branched into the joint capsule to form the retinacular artery. Among these, the posterior superior retinacular artery originated from the medial circumflex femoral artery in 10 hip joints, and hips originated from the branch of the IGA (absence of MFCA). Whether it originated from the MFCA or IGA, the origin of the posterior superior retinacular artery was basically constant (Fig. 9). The point was in the upper part of the bone groove, and the nearest distance to the bone groove was 1.11 ± 0.96 cm. Therefore, the entry point of the bone groove would not damage the deep branch of the medial circumflex femoral artery and its posterior superior retinacular artery, and there was an approximately 1 cm safe space for drilling the bone groove and implanting it into the quadratus femoris muscle pedicle bone flap (Fig. 10). In addition, the articular capsule can also be used as a reference for drilling bone grooves. This anatomical structure provides a basis for preserving the posterior superior support of the quadratus femoris muscle pedicle bone flap.

### Clinical application

**Fig. 11:**
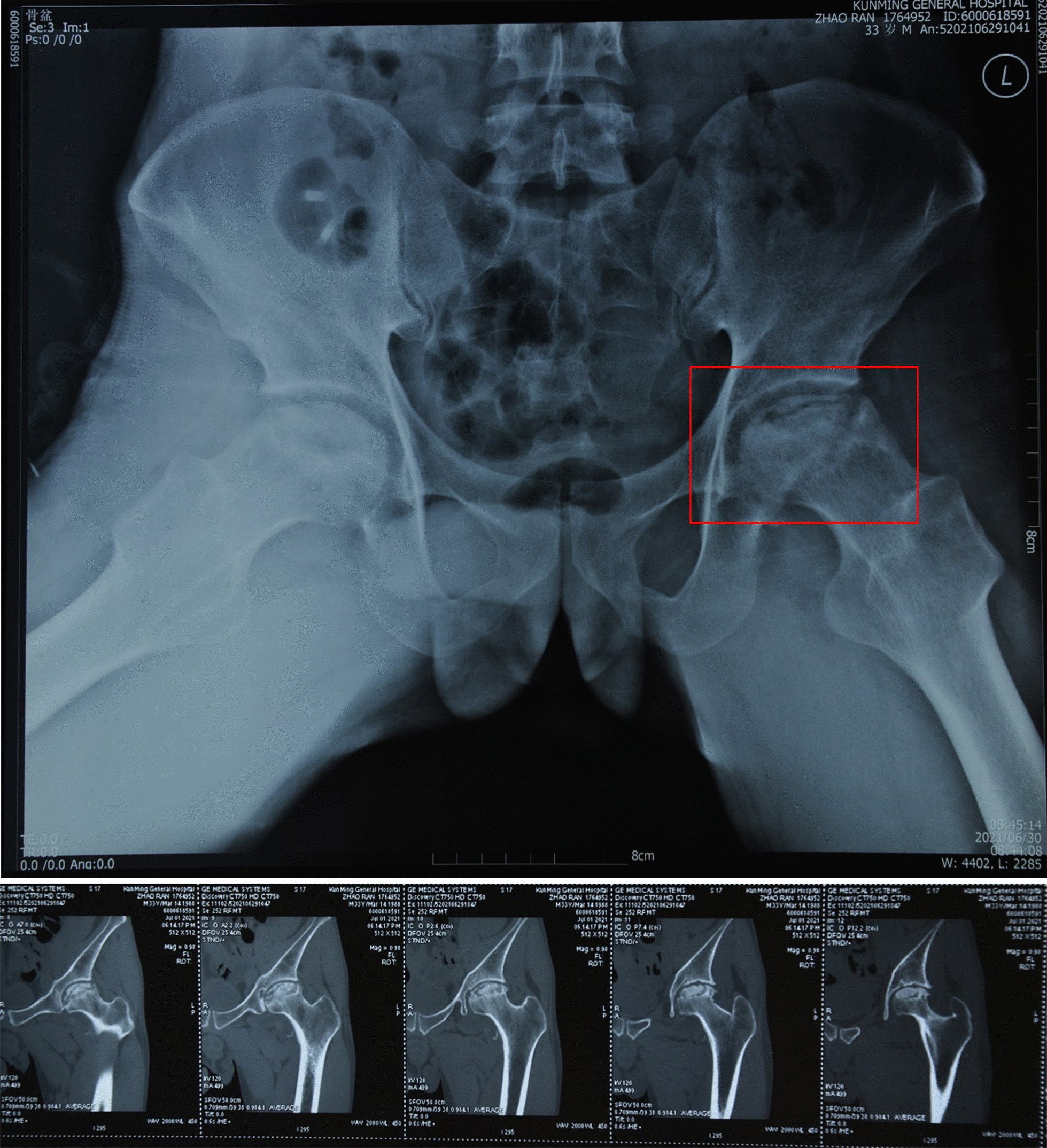
Imaging examination of the patient on admission. Red box: The necrotic area of the femoral head, ARCO stage III

We are already attempting to use this kind of surgery in the clinic. In this study, a patient with ONFH was treated with a modified quadratus femoris muscle pedicle bone flap. The patient was hospitalized with left hip pain and an abnormal gait. An X-ray examination was performed after admission and indicated bilateral ONFH; the left side was stage III (Fig. 11).

**Fig. 12:**
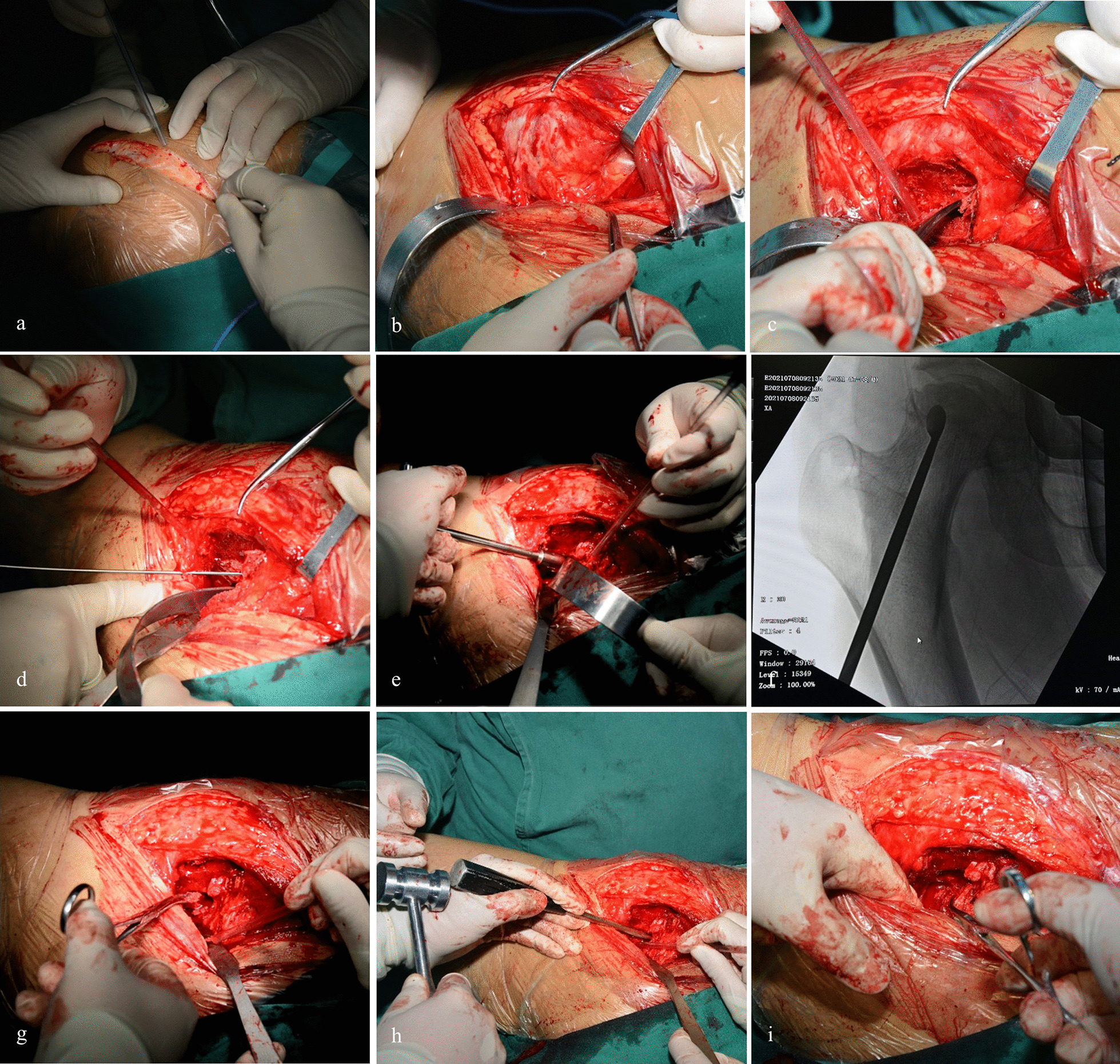
Surgical procedure of the modified quadratus femoris bone graft. **a** Posterior hip approach; **b** Exposure of the quadratus femoris muscle; **c** Chiseling of the quadratus femoris bone flap. **d** Drilling into a Kirschner needle to mark the position of the bone groove; **e** Drilling of a bone slot with a ring saw; **f** Removal of dead bones around the groove. **g** Implantation of allogeneic bone; **h** Compaction of cancellous bone. **i** Embedding of the bone flap into the bone groove

Before the operation, digital subtraction angiography (DSA) was performed to determine the presence of the posterior superior retinacular artery. We used the posterior surgical approach. Blunt separation of the gluteus medius muscle was performed to expose the quadratus femoris muscle. The quadratus femoris muscle pedicle bone flap was chiseled and loosened properly. The location of the drill hole is chosen at the intertrochanteric insertion point near the hip joint capsule. The operation within this safe distance would not damage the most important source of blood of the femoral head. The necrotic area was further cleared of dead bone after drilling. Partial autologous or allogeneic bone was transplanted into the bone groove and moderately compacted the bone. Then, the bone flap was inserted into the bone slot, and pressure was applied to make the flap more firmly inserted. C-arm fluoroscopy was used to examine whether the bone flap reached the necrotic area. Then, the area was checked again to determine whether the bone flap was firmly inserted. After layer-by-layer suturing, the surgical wound was closed (Fig. 12).

**Fig. 13:**
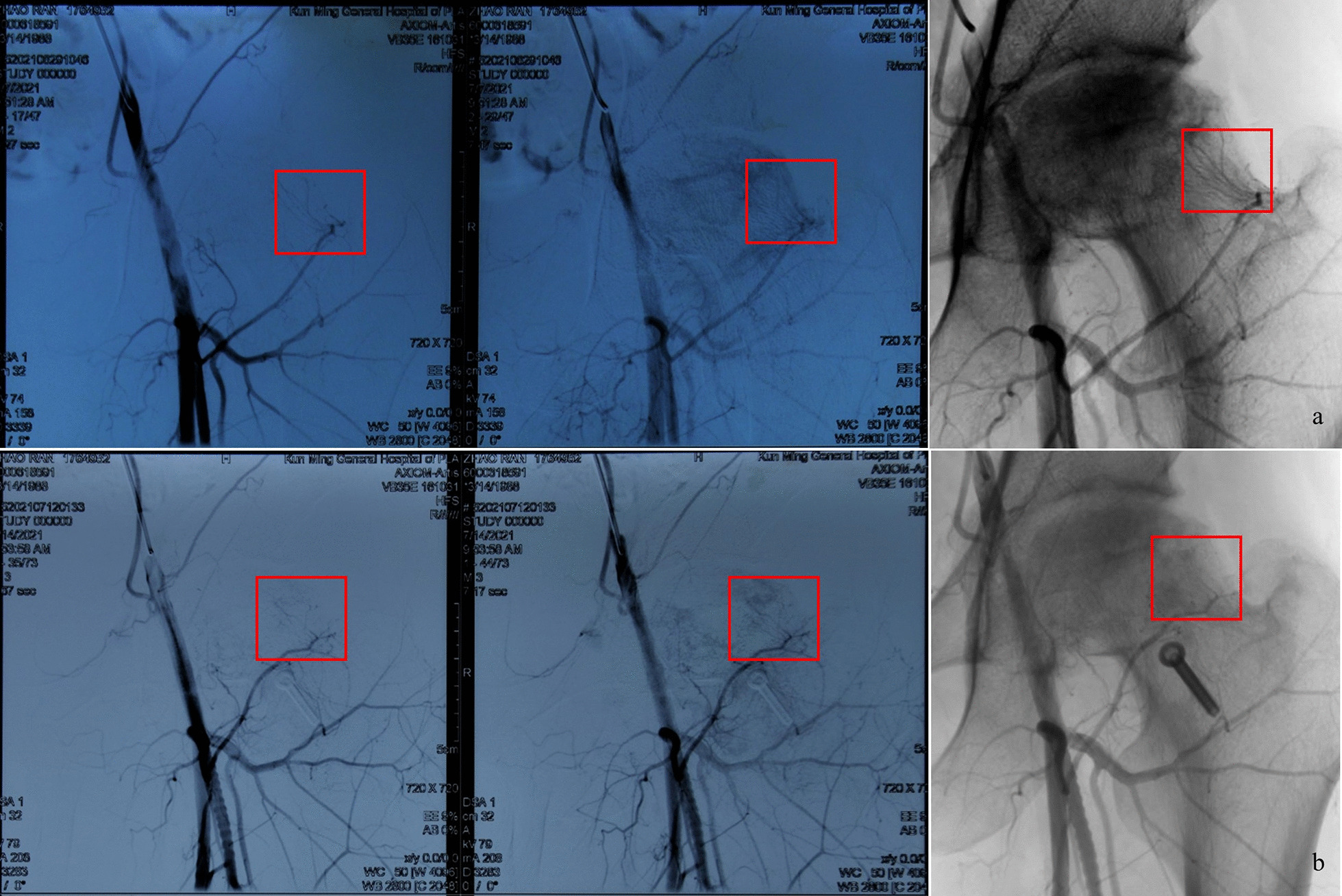
Preoperative identification of the posterior superior retinacular artery by DSA. **a** PRE-operation; **b** post-operation

Next, the bone flap was fixed firmly with 1 screw, and after washing, the operative area was sutured layer by layer. DSA examination before and after the operation showed that there was no injury to the posterior superior retinacular artery (Fig. 13).

**Fig. 14:**
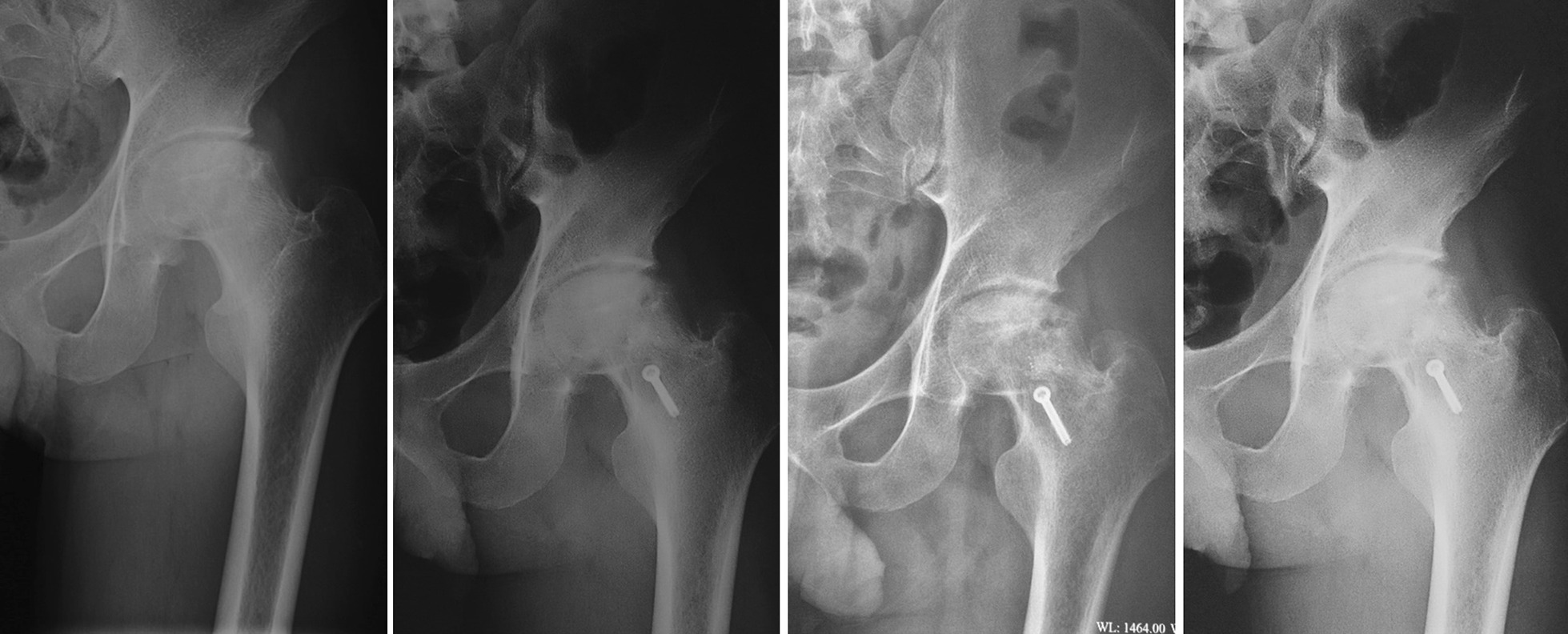
X-ray comparison of patients before and after the operation

Postoperative X-ray showed that the dome of the flattened femoral head had been partially corrected. The traces of the embedded bone flap were clearly visible. One month and three months after the operation, the bone flap was firmly embedded and did not move, and the bone flap was partially fused with the surrounding bone (Fig. 14).

## Discussion

The pathogenesis of ONFH is considered to involve damage of the blood supply to the femoral head due to blockage of the blood flow in the femoral head, eventually resulting in osteonecrosis [7]. Currently, ARCO staging is often used in the staging of ONFH. The vast majority of participants are in favor of the staging method presented in the revised ARCO staging system in 2019 [8]. For patients with early ONFH, including ARCO I and II, core decompression can achieve satisfactory clinical results, and the recommended grade is grade A (indicating good evidence) [2]. However, for ARCO III stage patients and even some ARCO II stage patients, core decompression alone has difficulty achieving satisfactory results, and necrosis will continue to progress, resulting in hip arthropathy and the need to undergo hip arthroplasty [9, 10].

Vascular pedicled bone grafts are grade B (fair evidence) in the recommended treatment of ONFH and are recognized as an effective methods [2]. The purpose of vascularized bone grafts is to remove necrotic bone from the femoral head and replace it with viable, structurally sound bone. This operation is mainly divided into two kinds: local pedicle bone grafts and free vascularized bone grafts. A quadratus femoris pedicle bone graft is a kind of local pedicle bone graft that was first described by Judet [11] in 1962 to treat patients with femoral neck fracture and was proven to achieve good clinical results. Later, quadratus femoris pedicle bone grafts were gradually applied for the treatment of femoral head necrosis. Research shows that the quadratus femoris pedicle bone graft can achieve good clinical results for patients with ARCO stage II disease but that the outcomes are poor for patients with stage III or IV disease, who usually cannot avoid final total hip arthroplasty (THA). Free vascularized fibula grafts (FVFGs) are widely used in free vascularized bone graft operations. Currently, FVFGs are generally believed to be applicable to patients with ARCO stage III or IV disease and can delay or even avoid THA [12]. However, this operation also causes local defects of the fibula and requires vascular anastomosis during the surgery, which increases the time and difficulty of the operation. In the past, the effect of the quadrate femoris bone flap in patients with ARCO III or IV was not ideal. The reason is that the blood supply is not as precise as that of the FVFG because the surgical method destroys the residual blood supply of the femoral head. In this study, it was found that the posterior superior retinacular artery originated from the MFCA of the circumflex femoral artery ring and reached the femoral head along the posterior and upward direction of the femoral neck. Our findings are basically consistent with those of other scholars [13–15]. The reasons for the poor effect of quadratus femoris pedicle grafts are not only the more serious factors of ONFH but also the disadvantages of the operation itself. In quadratus femoris pedicle bone grafts, a "T" incision is required in the hip capsule, which inevitably leads to injury of the MFCA branches, especially the posterior superior supporting artery, the most important feeding artery of the femoral head. Once such vessels are damaged, they aggravate the ischemic state of the femoral head. Although pedicle bone grafts can provide partial blood supply to the femoral head, it is difficult to compensate for the adverse effects caused by injury to the posterior superior supporting artery of the femoral head. This limits the use of the quadratus femoris bone flap graft.

Quadratus femoris pedicle bone grafts involve a vascularized pedicle bone graft method, and we improved this approach and created a new technique for the operation. The new method retains the integrity of the posterior superior retinacular artery of the femoral head on the basis of the rich blood supply of the quadratus femoris pedicle bone flap. There is no need to incise the hip joint capsule during the operation, which significantly reduces the surgical time and the difficulty of the operation. In the new technique, after the quadratus femoris muscle pedicle bone flap was chiseled, a bone groove was drilled in the upper and inner parts of the chisel position towards the outer upper quadrant of the femoral head.

The results of the experiment revealed that under the premise of fully drilling the bone groove to reach the necrotic area, the quadratus femoris muscle pedicle bone flap still has enough length and migration distance to be firmly embedded in the bone groove, which can provide the exact blood supply to the necrotic area of the femoral head. To increase the migration distance of the quadratus femoris muscle pedicle bone flap, it is necessary to release the quadratus femoris muscle and to be careful not to damage the blood vessels on the fascial surface of the quadratus femoris muscle. The longer migration distance of the bone flap can not only make it more easily embedded into a deeper position of the femoral head but also reduce the traction of the quadratus femoris muscle to the bone flap.

The greatest innovation of the new technique is to retain the integrity of the posterior superior retinacular artery of the femoral head. The previous transposition of the quadratus femoris muscle pedicle bone flap requires a "T" incision of the hip joint capsule, and the artery will be injured. Our technique does not require incision of the hip joint capsule. We can preserve the integrity of the blood supply of the femoral head, such as the MFCA and its branches. The current consensus is that the MFCA is the most important blood-supply artery of the femoral head; it ascends around the femoral neck and sends a branch into the hip joint capsule at the posterior upper portion of the femoral neck fundus. This branch is the posterior superior retinacular artery, which is considered to be the most important source of blood for the femoral head. In the process of drilling the bone groove, an improper location of the entrance of the bone groove can easily damage the MFCA and the posterior superior retinacular artery. We believe that it is safe to drill a hole in the intertrochanteric position outside of the hip joint capsule behind the femur.

Our study has some limitations. First, only 6 cadavers (12 hips) were included. Second, further clinical research needs to be carried out, with the collection of more cases and a longer follow-up period to analyze the therapeutic outcomes. Third, all the subjects were Chinese, which may have introduced ethnic bias.

## Conclusion

We confirmed the feasibility and safety of modified quadratus femoris muscle pedicle bone grafts through anatomical study, and we also verified this point through clinical practice. This modified procedure reduces the surgical time and the amount of bleeding, reduces the difficulty of the operation, retains more blood supply of the femoral head, and speeds up the postoperative recovery of patients. This modified method can be used in patients with stage II or III ONFH and may achieve satisfactory clinical results.

## Data Availability

The datasets used and/or analysed during the current study are available from the corresponding author on reasonable request.
